# Enhanced Mechanical Properties of Cast Cu-10 wt%Fe Alloy via Single-Pass Friction Stir Processing

**DOI:** 10.3390/ma16217057

**Published:** 2023-11-06

**Authors:** Xiaobo Yuan, Hui Wang, Ruilin Lai, Yunping Li

**Affiliations:** 1 State Key Lab for Powder Metallurgy, Central South University, Changsha 410083, China; yuanxiaobo@csu.edu.cn (X.Y.);; 2 Research Institute of Light Alloy, Central South University, Changsha 410083, China

**Keywords:** Cu-Fe alloy, friction stir processing, microstructure, mechanical properties

## Abstract

In this study, Cu-10 wt% Fe alloy in as-cast state was modified using friction stir processing (FSP). The microstructure evolution of Cu-10 wt% Fe alloys in different states was characterized in detail using scanning electron microscopy (SEM), electron backscatter diffraction (EBSD) and transmission electron microscopy (TEM). The results show that due to dynamic recrystallization, the FSPed Cu-10 wt% Fe alloy obtained a uniformly equiaxed ultrafine microstructure with low density of dislocation, high proportion of high-angle grain boundaries (HAGBs), and high degree of recrystallization. Fine equiaxed grains with an average size of 0.6 μm were produced after FSP. Many fine-precipitate Fe-phases with an average size of 20 nm were uniformly distributed in the Cu matrix. The FSPed samples possessed excellent mechanical properties, such as high Vickers hardness (163.5 HV), ultimate tensile strength (538.5 MPa), and good elongation (16%). This single-pass FSP method does not require subsequent aging treatment and provides a simple and efficient way to improve the properties of Cu-Fe alloys.

## 1. Introduction

With the emergence of the 5G era, there is a growing demand for a Cu-Fe alloy due to its abundant raw materials, low cost, and excellent electromagnetic shielding performance [1,2,3]. By incorporating a high concentration of Fe (more than 4 wt%), it is possible to obtain a Cu-Fe alloy with outstanding mechanical properties and electromagnetic shielding properties [4]. However, it is important to note that Cu-Fe alloy, being a typical metastable immiscible system, often experiences liquid–liquid phase separation (LLPS) during the solidification process [5,6]. This phenomenon leads to an uneven distribution of the Fe phase, particularly in Cu-Fe alloys with high Fe content, resulting in a significant deterioration of the mechanical properties of the Cu-Fe alloys [7,8,9].

It has been reported in the publications that the addition of minor alloying elements to immiscible Cu-Fe alloys is an effective method to improve the wettability of the liquid-phase interface [10]. Recent studies have shown that the addition of Ag [11,12], Mg [9], Nb [13,14], Zr [15], Co [16], Si [17,18], etc., to Cu-Fe alloys can refine the microstructure and improve comprehensive performance. Zhang et al. [11] reported that the interfacial energy of Cu-Fe alloy was reduced and the nucleation rate of the Fe phase was increased by adding 0.5 wt % Ag. Yuan et al. [9] showed that the addition of magnesium to the Cu-6.5Fe-Mg alloy suppressed the dendritic segregation and coarsening of the Fe phase, thus significantly improving the mechanical properties. The results reported by Ding et al. indicate that the addition of Nb to Cu-10 wt% Fe alloys is favorable for inducing the generation of Fe_2_Nb phases and promoting heterogeneous nucleation of Fe-rich phases [13]. Although the addition of minor alloying elements to Cu-Fe alloys has been well understood, the excellent performance is accompanied by the exploration of a complex deformation process and cumbersome heat treatment process when adding different alloying elements. There were efforts to apply severe plastic deformation (SPD) to Cu alloys, using methods such as friction stir processing (FSP) [19] and equal channel angular pressing (ECAP) [20]; high pressure torsion (HPT) [21] is also an effective means to improve the comprehensive properties.

FSP presents promise as an SPD method capable of producing equiaxed ultrafine-grained (UFG) structures with more closely matched strength and plasticity [22]. This method is usually employed for modifying surface microstructures. Wang et al. [23] reported that FSP markedly decreased the size of the precipitated phase in Cu-0.83Cr-0.14Zr alloy to 3.1 nm, leading to an improvement in both the mechanical and electrical conductivity of the alloy. Likewise, Naik et al. [19] observed similar outcomes when FSP was applied to Cu-0.62Cr-0.11Zr alloy. Meanwhile, Escobar et al. [24] used FSP on Cu-4Nb alloy and discovered that the Nb particles were finely refined, altering the recrystallisation mechanism of Cu-4Nb alloy. FSP has demonstrated the capacity to significantly enhance the properties of Cu alloys; however, it has yet to be investigated whether FSP can enhance the strength of Cu-Fe alloys.

In the present investigation, a cast Cu-Fe alloy underwent single-pass FSP for the first time. The study comprehensively examines microstructure evolution, encompassing grain size and precipitation, and its impact on the hardness and tensile properties of the Cu-Fe alloy. The objective of this research is to enhance the mechanical properties of Cu-Fe alloys using a simple single-pass FSP.

## 2. Materials and Methods

### 2.1. Sample Preparation

In this study, a 6 mm cast Cu-10 wt% Fe alloy plate (referred to as as-cast) was utilized, containing a nominal composition of 10 wt% Fe and a residual amount of Cu. To enable a comparison with FSP, a segment of the as-cast Cu-10 wt% Fe alloy was cold-rolled and deformed at room temperature, exhibiting a total reduction of 20%. Meanwhile, the remaining part of the as-cast Cu-10 wt% Fe alloy was subjected to single-pass friction stir processing (named FSPed), by means of a CFSW machine (CFSW-B800, CFSW, Beijing, China) at 500 rpm with a consistent travelling speed of 30 mm/min. To reduce grain coarsening due to high heat input, water cooling is applied behind the moving rotary tool. The stirring tool utilized was an H13 steel tool with a shoulder diameter of only 12 mm, a taper pin length of 5 mm, and a root diameter of 4.2 mm. FSP experiments were performed in an air atmosphere.

### 2.2. Microstructure Characterization

The optical microscope (OM, Lecia DM4000, Wetzlar, Germany) was adopted to characterize the macrostructural feature of the Cu-10 wt% Fe alloy. The microstructure was scrutinized and examined employing a field-emission scanning electron microscope (FESEM, Quanta 650 FEG, FEI, Boston, MA, USA) outfitted with electron backscatter diffraction (EBSD, Oxford, AZtecCrystal2.12, Oxford, UK) and a field-emission transmission electron microscope (FETEM, FEI Tecnai G2 F20, Boston, MA, USA) with an operating voltage of 200 kV. The OM samples were ground and mechanically polished before being etched in a solution comprising 20 mL hydrochloric acid, 16 g iron (III) chloride, 100 mL distilled water, and 100 mL ethyl alcohol at room temperature. After the completion of grinding and mechanical polishing, the specimens for EBSD observation were electropolished at a direct current of 5 V in a solution of 2.5 g carbamide, 25 mL isopropyl alcohol, 125 mL phosphoric acid, 135 mL ethyl alcohol and 250 mL distilled water, at −10 °C. The TEM thin foils, with a diameter of 3 mm, were prepared utilizing the electrolytic double spray technique in a solution of 200 mL nitric acid and 400 mL methanol at approximately −30 °C. 

### 2.3. Mechanical Assessment

**Figure 1:**
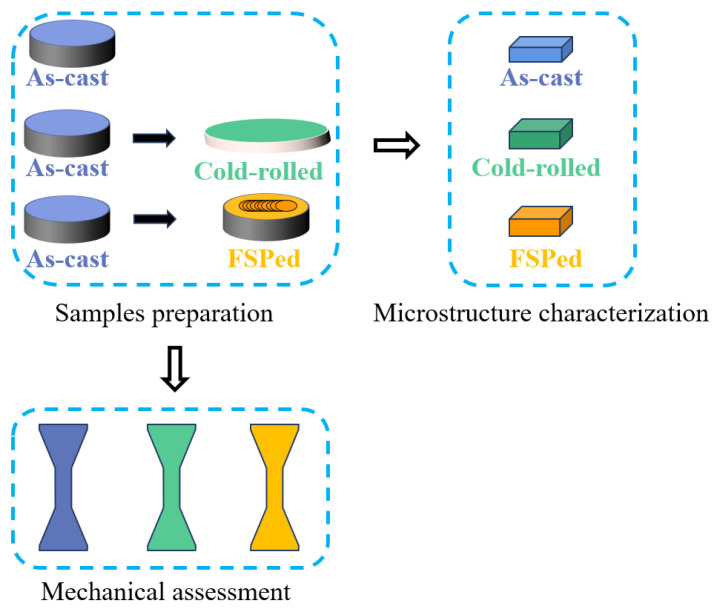
Illustration of the experimental flow.

Dog-bone-shaped tensile specimens with a gauge length of 8 mm and cross-section area of 3.4 mm × 2 mm were prepared. Tensile testing was conducted under room temperature conditions using a universal testing machine (UTM 5105, SUNS, Shenzhen, Guangdong, China), following the standard of GB/T 228.1-2021 [25] with a strain rate of 1.0 × 10^−3^ s^−1^. Measurements were performed at least three times to ensure the accuracy of the tensile tests. Vickers hardness tests were conducted using a microhardness tester (200HV-5, HUAYIN, Laizhou, Shandong, China), applying a load of 1 kG for a duration of 10 s. The cold-rolled samples used for observation and mechanical property testing were cut along the rolling direction. FSPed samples used for observation and mechanical property testing were all cut perpendicular to the processing forward direction, and contain only the weld nugget zone. A graphical illustration of the experimental flow is displayed in Figure 1.

## 3. Results

**Figure 2:**
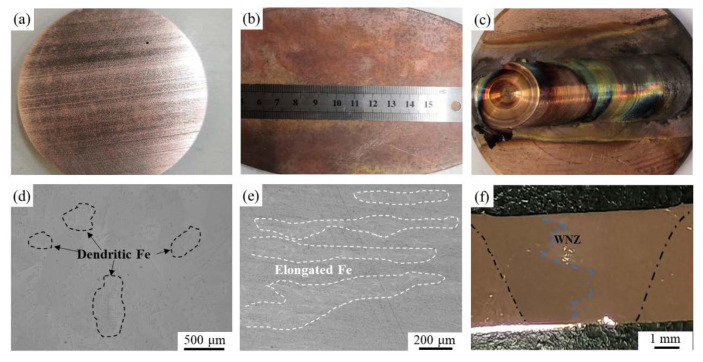
Macrostructure evolutions of Cu-10Fe alloy in different conditions: (**a**,**d**) as-cast sample, (**b**,**e**) cold-rolled sample, and (**c**,**f**) FSPed sample.

Figure 2 displays macroscopic images of Cu-10 wt% Fe alloy specimens in three different conditions: as-cast, cold-rolled, and FSPed. As illustrated in Figure 2c, the sample’s surface turned black and exhibited onion rings and keyholes after following single-pass FSP; these are typical macroscopic morphologies of Cu alloys following FSP [26]. Specifically, Figure 2d shows the typical two-phase dendritic structure of the as-cast Cu-10 wt% Fe alloy, featuring incipient Fe-rich dendrites (marked with a black dashed box) and the matrix. Figure 2e displays the metallographic microstructure of the cold-rolled Cu-10 wt% Fe alloy, revealing that the dendritic Fe phases in the alloy transform into elongated fibers (marked with a white dashed box) after cold rolling. In contrast, Figure 2f indicates that the weld nugget zone shows significant material mixing after FSP. This is because high rotational speed leads to increased heat input and improved flow behavior of the material. 

**Figure 3:**
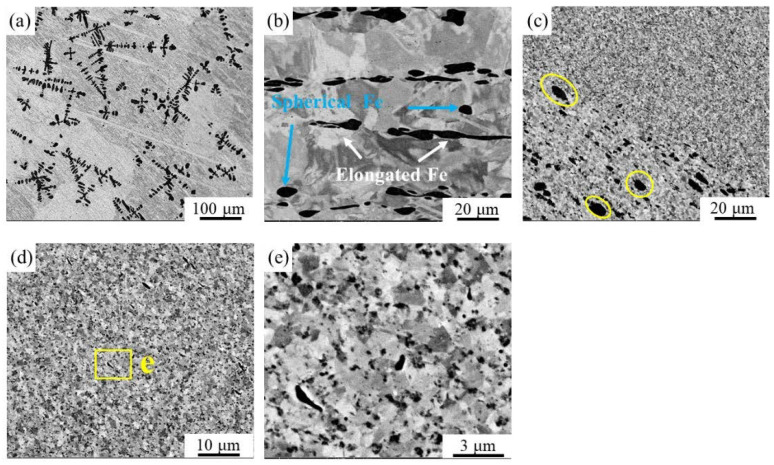
Microstructure evolutions of Cu-10Fe alloy in different conditions: (**a**) as-cast sample, (**b**) cold-rolled sample, (**c**) TMAZ of FSPed sample, and (**d**,**e**) WNZ of FSPed sample.

The SEM figures of Cu-10 wt% Fe alloy in the as-cast, cold-rolled, and FSPed conditions, observed using the backscattered electron image model, are shown in Figure 3. The black area signifies the Fe phases, while the light gray area represents the Cu matrix. The micrograph as shown in Figure 3a clearly reveals a large number of dendritic and spherical Fe phases within the as-cast Cu-10 wt% Fe alloys. These features are more finely dimensioned than the dendritic Fe phases evident in Figure 2d. After undergoing a 20% reduction cold-rolling process, it is visible in Figure 3b that in the dendritic Fe phases obvious plastic deformation occurred along the rolling direction and elongated into a fiber structure (white arrows), while the spherical Fe phase experienced less deformation (blue arrows). The single-pass FSPed Cu-10 wt% Fe alloy has resulted in the refinement of both the Cu matrix and dendritic Fe phases, due to intense deformation in the weld nugget zone, as depicted in Figure 3c–e. The yellow circle in Figure 3c shows the area not stirred by the stirring pin in the thermomechanical affected zone, where the Fe phase is deformed but still coarse grained. A more closely magnified image in the weld nugget zone is exhibited in Figure 3e, indicating that the Fe phases were refined and evenly dispersed throughout the Cu matrix.

**Figure 4:**
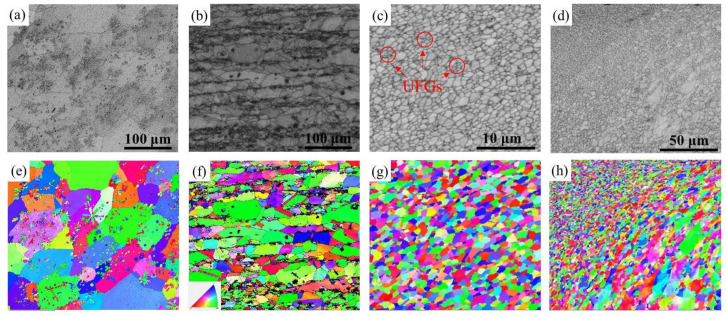
Band-contrast (BC) maps and corresponding inverse-pole-figure (IPF) maps of Cu-10Fe alloy in different conditions: (**a**,**e**) as-cast sample, (**b**,**f**) cold-rolled sample, (**c**,**g**) weld nugget zone of FSPed sample, and (**d**,**h**) thermomechanical affected zone of FSPed sample.

**Figure 5:**
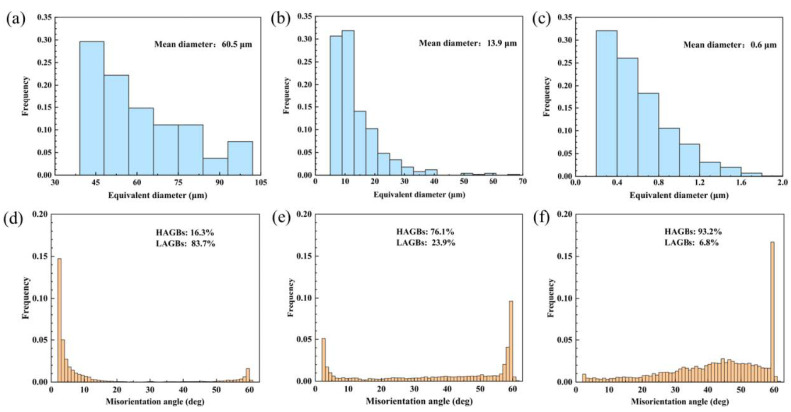
EBSD images of grain size distribution (in blue) and grain boundary misorientation distributions (in yellow) of (**a**,**d**) as-cast sample, (**b**,**e**) cold-rolled sample, and (**c**,**f**) FSPed sample.

The EBSD analysis revealed the microstructures of the three samples. Figure 4 displays the band contrast (BC) maps and corresponding inverse pole figure (IPF) of the Cu-10 wt% Fe alloy under as-cast, cold-rolled, and FSPed conditions. Statistically acquired maps of grain boundary orientation distribution and grain size distribution of Cu grain for Cu-10 wt% Fe alloy under three conditions can be found in Figure 5. High-angle grain boundaries (HAGBs) are defined as having an orientation difference of angle ≥15°, while low-angle grain boundaries (LAGBs) are defined as having an orientation difference of 2 ≤ angle < 15°, respectively. The EBSD results indicate that the microstructure of the as-cast samples comprises coarse Cu grains with a mean size of 60.5 μm. The cold-rolled samples showed that their HAGB proportion was relatively low, accounting for only 16.3%. After deformation, the cold-rolled samples underwent recrystallization; the percentage of HAGBs increased to 76.1%, and the grains were refined to 13.9 μm. The HAGB proportion in the FSPed sample was exceptionally high at 93.2%, indicating that recrystallization was almost accomplished. Additionally, the Cu grains of WNZ became significantly refined, and consist of equiaxial ultrafine grain (UFGs) with grain size of 0.6 μm, significantly smaller than that of the as-cast and cold-rolled samples. The BC map and IPF map show that the FSPed sample is characterized by a typical equiaxial recrystallized microstructure without any significant texture.

**Figure 6:**
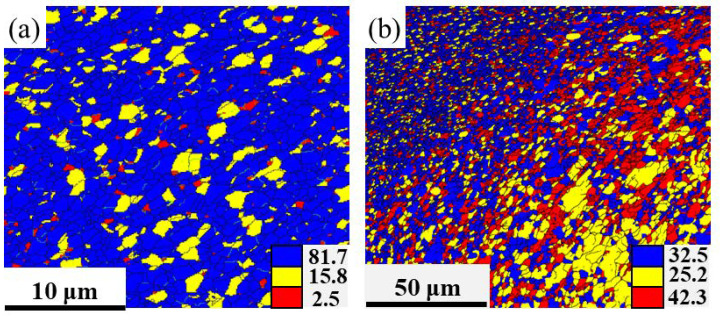
Distribution maps of recrystallized (in blue), substructured (in yellow), and deformed (in red) grains, (**a**) weld nugget zone and (**b**) thermomechanical affected zone.

The distribution and area percentage of recrystallized (in blue), substructured (in yellow), and deformed (in red) grains for both the weld nugget zone and thermomechanical affected zone in the FSPed samples are displayed in Figure 6a,b. The FSPed specimen for the weld nugget zone displays an ultrafine equiaxed crystalline arrangement, with the recrystallized grain fraction reaching 81.7%. The percentage of recrystallized grains in the thermomechanical affected zone has decreased by only 32.5%, which can be attributed to the thermomechanical affected zone being only partially composed of a weld nugget zone. Cu is a stacking-fault energy (SFE) material, and when deformed at temperatures above 0.5 times its melting point, discontinuous dynamic recrystallization (dDRX) takes place [27]. This results in the formation of new grains that are surrounded by high-angle boundaries, due to the nucleation of grain boundary irregularities and subsequent growth [27]. This is supported by the presence of 93.2% HAGBs, as seen in Figure 5f.

**Figure 7:**
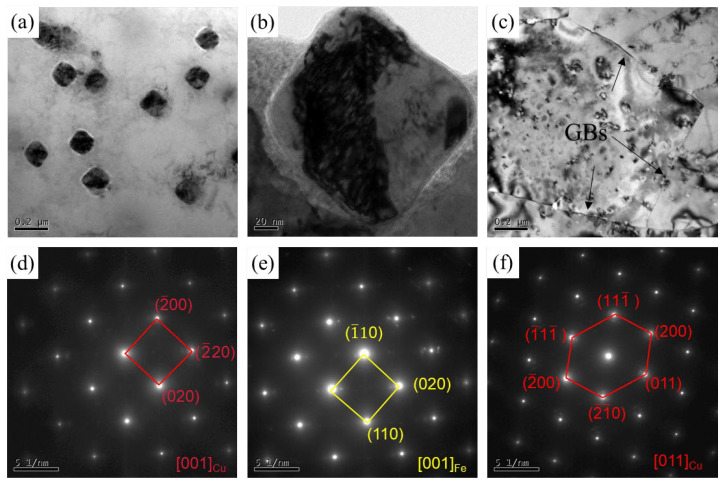
(**a**–**c**) Bright-field TEM images and (**d**–**f**) corresponding SAED of (**a**,**b**,**d**,**e**) as-cast sample and (**c**,**f**) FSPed sample.

**Figure 8:**
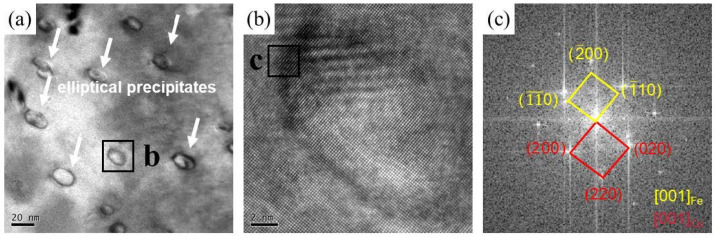
(**a**,**b**) The high-resolution TEM images and (**c**) the corresponding FFT pattern of FSPed Cu-10Fe alloy.

Further high-resolution characterization such as TEM is necessary due to the limited information provided by SEM and EBSD. Figure 7 includes bright-field TEM images and corresponding selected area electron diffraction (SAED) of both as-cast samples and FSPed samples. Figure 7a illustrates the morphology, showing independently distributed square particles within the matrix in the as-cast sample. According to the SEAD results illustrated in Figure 7d,e, it is verified that the matrix is composed of Cu, whereas the square particles contain Fe phases. Conversely, the particles in the FSPed specimen exhibit dispersed distribution features and are considerably smaller in size. Figure 8 presents the high-resolution TEM (HRTEM) images of the Cu-10 wt% Fe alloy processed using FSP along with its corresponding fast Fourier transform (FFT) patterns. Based on the SAED image in Figure 8c, it can be concluded that these fine-precipitate phases diffusely distributed in the Cu matrix with an average size of about 20 nm are also Fe phases.

**Figure 9:**
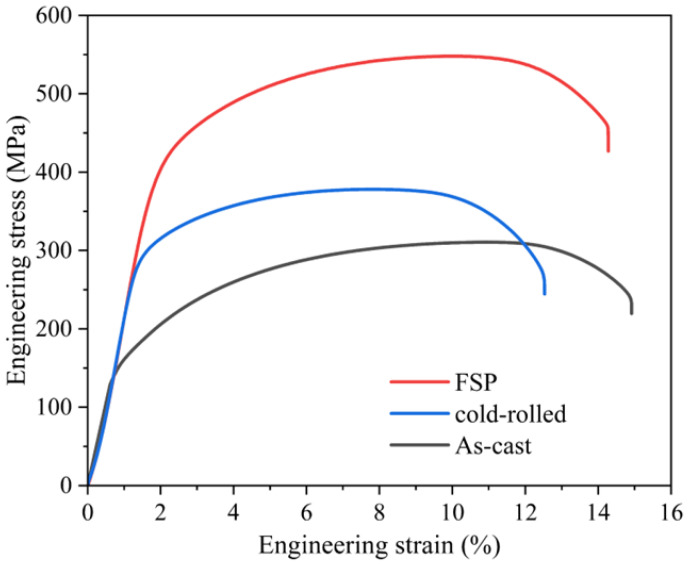
Tensile stress–strain curves of Cu-10Fe alloy of three specimens.

**Figure 10:**
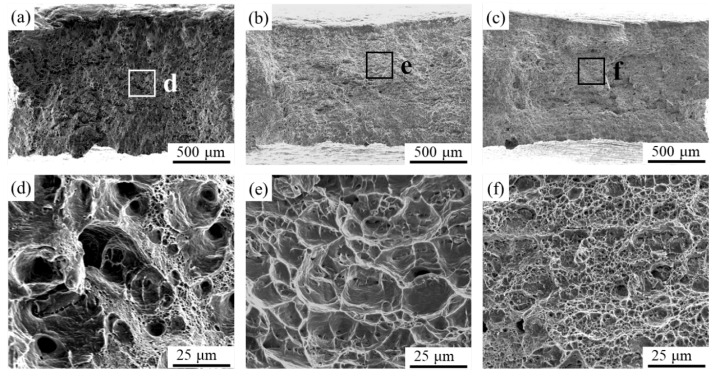
Fracture morphologies of Cu-10Fe alloy in different conditions: (**a**,**d**) as-cast sample, (**b**,**e**) cold-rolled sample, and (**c**,**f**) FSPed sample.

The stress–strain curves of three specimens are presented in Figure 9, and their mechanical properties are summarized in Table 1. In the case of the as-cast Cu-10 wt% Fe alloy, the ultimate tensile strength (UTS) and elongation at fracture (EF) were 303.7 MPa and 16.6%, respectively. The UTS of the cold-rolled specimens increased to 373.6 MPa compared to the cast Cu-10 wt% Fe alloy, whereas the EF decreased to 12.1%. The mechanical properties demonstrate a more significant increase in FSP as the degree of deformation increases. The UTS and EF increased to 538.3 MPa and 16.1%, respectively. The microhardness shows a consistent pattern of change with UTS. In its as-cast state, Cu-10 wt% Fe alloy has an average Vickers hardness of 87.5 HV. However, the Vickers hardness of Cu-10 wt% Fe alloy increases to 114.9 HV after being subjected to cold-rolling deformation. It is apparent that the microhardness of WNZ of FSPed samples is remarkably high, at 163.5 HV. Figure 10a–f provides the corresponding morphology of the fracture, which displays significant necking. In the high-magnification images, numerous typical microscopic toughness features are observable. These features indicate that the fracture was ductile in nature.

## 4. Discussion

FSP is an effective method to improve the mechanical properties and grain refinement of Cu-10 wt% Fe alloys. In this work, Cu-10 wt% Fe alloys with a UTS of 538.3 MPa, an EF of 16.1%, and an average grain size of 0.6 µm were prepared using FSP at a low rotational speed (seen in Table 1). It is shown that the action of FSP during the preparation process has a significant effect on the microstructure evolution of Cu-10 wt% Fe alloys.

During solidification, Fe precipitates out of the Cu-rich liquid phase to form nuclei composed of the Fe phase. The growth of these Fe-phase nuclei necessitates the absorption of Fe atoms found in the Cu liquid [5,28]. However, as these nuclei expand to a certain size, the Cu liquid surrounding them is unable to sustain their growth due to insufficient Fe content. The growth of these Fe-phase nuclei necessitates the absorption of Fe atoms found in the Cu liquid. As the temperature of the Cu liquid drops, dendritic formations of Fe-phase cells emerge that are evenly dispersed throughout the Cu matrix [28,29]. As shown in Figure 3a, most of the iron forms dendrites, while the remaining portion dissolves in the matrix in the solid-solution form.

After cold rolling the Cu-10 wt% Fe alloy, the small Fe phase with a spherical shape and a size range of 5–20 mm undergoes minimal deformation (blue arrows in Figure 3b). In contrast, the dendritic Fe phase elongates along the rolling direction, creating a fibrous structure, presented by white arrows in Figure 3b. This grain microstructure is also a typical feature of cold-rolled Cu-Fe alloys reported in the published literature [29,30], with the presence of larger-sized fibrous Fe phases due to the lower reduction used in this study. The fine Fe-phase dendrites are susceptible to large stress concentrations, due to plastic deformation, mainly at the dendrite tips, resulting in the formation of a fibrous Fe phase along the rolling direction [31]. After undergoing cold rolling, the applied stress generated by the rolling process surpasses the yield strength of the Fe phase, leading to plastic deformation. In the cold-rolling process, an abundance of dislocation tangles, fine LAGBs, and fibrous tissue are formed within the Cu matrix along the direction of rolling [32]. 

Uniform equiaxed grains were obtained in FSPed samples, which was a result of the dynamic recrystallization (DRX) refining mechanism [24]. Histogram analysis of grain characteristics in Figure 5f demonstrates that DRX occurs in the weld nugget zone. This is indicated by a substantial increase in the frequency of high-angle grain boundaries, which are nascent DRX grains, and a significant decrease in the frequency of low-angle grain boundaries (LAGBs) relative to as-cast samples and cold-rolled samples. This is consistent with findings from previous studies and is attributed to the combination of frictional heat and mechanical agitation during FSP [24,33,34]. The finer equiaxed grains and higher percentage of HAGBs suggest that the fine-grained structure of incompletely recrystallized grains was retained in the FSPed samples, due to the relatively low heat input. Compared with the EBSD data of Cu-Cr-Zr alloys after FSP studied by Wang et al. [23], FSP can significantly refine the grains while maintaining a high percentage of HAGBs. Additionally, ultrafine Cu grains, ranging from 100 to 200 nm in size, appear within the weld nugget zone, as indicated by red circles in Figure 4c and Figure 7c. 

The bright-field TEM image in Figure 7c shows that the grain boundaries of the FSPed sample are relatively clear, sharp, and extremely straight. These typical features of HAGBs are consistent with the results reported in the literature [23,35]. In addition, a lower dislocation density and the fact that most of the GBs are HAGBs are observed in the equiaxed UFG structure of the FSPed sample, which is comparable to the results observed in the SPD UFG Cu-Cr-Zr alloy by Purcek et al. [21] and Lai et al. [36]. According to Bellon et al. [37], sustained high shear rates can stabilize supersaturated metastable solid solutions in immiscible systems like the Cu-Nb system. In addition, Cu-Fe is also an immiscible system wherein shear-induced excess vacancies boost elemental de-mixing by increasing diffusivity [38,39]. During FSP, the Fe phase experiences fragmentation, refinement, and redistribution throughout the weld nugget zone. Moreover, within the shear deformation region, Fe is redistributed within the ultrafine Cu grains as a supersaturated solid solution and as dispersed nano-sized particles [24]. This is evidenced by the nano-sized Fe phases (white arrows) in Figure 8a. 

The present Cu-10 wt% Fe alloy, subjected to FSP, demonstrated an average grain size of 0.6 μm. This is comparable to the grain size of FSPed pure Cu, which, under water-cooled conditions, was found to be 0.4 μm in a study conducted by Xue et al. [40]. Moreover, Su et al. [41] pointed out that FSP of pure copper resulted in elongated grains close to the needle extraction area under dry-ice cooling conditions. Importantly, the elongated grains quickly changed to an equiaxed grain structure as they passed through the FSP tool. In this study, we applied rapid water-cooling conditions after FSP. Despite the water-cooling rate not being as fast as dry ice, it still maintained a very fast cooling rate [42,43]. This rapid cooling is beneficial in suppressing Cu grain growth and obtaining the UFG microstructure. Moreover, the presence of nanoscale precipitates in the Cu-10 wt% Fe alloy is pivotal in the microstructure evolution. Figure 7a displays the uniform distribution of many 200 nm sized particles in the as-cast Cu matrix. However, in Figure 8a, elliptical precipitates around 20 nm shown by the white arrows were observed after FSP. The nanoprecipitates provide additional nucleation sites and act as strong barriers against dislocation movement during the FSP process, thereby decelerating the grain growth process [44]. Thus, in FSP Cu-10 wt% Fe alloys incorporating nanoprecipitates, it is possible to maintain the UFG structure with deformation-induced characteristics that vanish in conventional FSP pure copper [23].

The effect of FSP not only results in significant microstructural changes, but also in the mechanical properties. The tensile curves of FSPed Cu-10 wt% Fe samples show the typical characteristics of fine equiaxial grains of Cu alloys, i.e., high strength and acceptable homogeneous elongation. The reported results show that, due to the UFG with low dislocation density, high HAGB percentage, and weak texture, the FSP Cu strain-hardening capacity is high [45]. Similarly, the microstructure of the weld nugget zone in this study is also characterized by high HAGB percentage and low dislocation density (Figure 5f and Figure 7c). During tensile plastic deformation, dislocations can be efficiently stored near the HAGBs, which improves the strain hardening capacity and contributes to obtaining good plasticity [46]. The increase in microhardness is attributed to the change in microstructure. The average hardness value of the Cu-10 wt% Fe samples significantly increased, from 87.5 HV in the as-cast condition to 163.5 HV in the FSPed condition. The finer grains and retained nano-sized precipitates in the FSPed samples account for this increase.

## 5. Conclusions

In this study, with the aim of improving the mechanical properties while preserving the microstructure, we carried out friction stir processing (FSP) on a cast Cu-10 wt% Fe alloy under water-cooled conditions. In addition, we performed cold-rolled deformation on a cast Cu-10 wt% Fe alloy for comparison. We carefully examined the microstructure and mechanical properties of the three samples, and arrived at the following conclusions and findings:

1. The iron phase in the cast Cu-10 wt% Fe alloy was dendritic in the matrix, and the Cu matrix had coarse grains with a grain size of 60.5 μm. Cold rolling reduced the grain size to 13.9 μm. Subsequently applying fast-cooling FSP yielded an equiaxed ultrafine grain microstructure with grain refinement to 0.6 μm in the as-cast Cu-10 wt% Fe alloys.
2. Compared to the precipitates in the BM, which have an average size of 200 nm, the precipitates in the FSPed sample were refined to an average size of only 20 nm, and some of the precipitates dissolved into the matrix. The HRTEM results have shown that the precipitates remained coherent with the Cu matrix.
3. Furthermore, the FSPed specimens showed excellent mechanical properties, including high hardness (165.5 HV), high tensile strength (538.3 MPa), and good elongation (16.1%). The mechanical properties are considerably higher compared to other cast and cold-rolled states. The enhancement in mechanical properties of FSPed samples is mainly attributed to the significant grain refinement and retention of fine precipitates.

## Figures and Tables

**Table 1:** Summary of mechanical properties of three samples.

State	UTS (MPa)	EF (%)	Microhardness (HV)
as-cast	303.7 ± 7.1	16.6 ± 1.5	87.5 ± 5.7
cold-rolled	373.6 ± 5.7	12.1 ± 1.7	114.9 ± 4.6
FSPed	538.3 ± 12.7	16.1 ± 1.3	163.5 ± 12.7

## Data Availability

Data available on request from the authors.
